# A Rationale for Music Training to Enhance Executive Functions in Parkinson’s Disease: An Overview of the Problem

**DOI:** 10.3390/healthcare6020035

**Published:** 2018-04-22

**Authors:** Teresa Lesiuk, Jennifer A. Bugos, Brea Murakami

**Affiliations:** 1 Music Therapy, University of Miami, Frost School of Music 5499 San Amaro Dr., N306, Coral Gables, FL 33146, USA; 2 Music Education, University of South Florida, School of Music, 4202 E. Fowler Ave., MUS 101, Tampa, FL 33620, USA; bugosj@usf.edu; 3 Department of Music, Pacific University, Forest Grove, OR 97116, USA; brea@ymail.com

**Keywords:** Parkinson’s Disease, executive functions, music training, fine motor, cerebellar-thalamocortical network (CTC)

## Abstract

Music listening interventions such as Rhythmic Auditory Stimulation can improve mobility, balance, and gait in Parkinson’s Disease (PD). Yet, the impact of music training on executive functions is not yet known. Deficits in executive functions (e.g., attention, processing speed) in patients with PD result in gait interference, deficits in emotional processing, loss of functional capacity (e.g., intellectual activity, social participation), and reduced quality of life. The model of temporal prediction and timing suggests two networks collectively contribute to movement generation and execution: the basal ganglia-thalamocortical network (BGTC) and the cerebellar-thalamocortical network (CTC). Due to decreases in dopamine responsible for the disruption of the BGTC network in adults with PD, it is hypothesized that rhythmic auditory cues assist patients through recruiting an alternate network, the CTC, which extends to the supplementary motor areas (SMA) and the frontal cortices. In piano training, fine motor finger movements activate the cerebellum and SMA, thereby exercising the CTC network. We hypothesize that exercising the CTC network through music training will contribute to enhanced executive functions. Previous research suggested that music training enhances cognitive performance (i.e., working memory and processing speed) in healthy adults and adults with cognitive impairments. This review and rationale provides support for the use of music training to enhance cognitive outcomes in patients with Parkinson’s Disease (PD).

## 1. A Rationale for Music Training to Enhance Executive Function in Parkinson’s Disease: An Overview of the Problem

Parkinson’s Disease (PD), a neurodegenerative disorder that affects more than five million adults in the U.S. alone, is often accompanied by deficits in executive functions in addition to motor symptoms such as bradykinesia, tremors, rigidity, and gait and postural difficulties [1]. Deficits in executive functions (e.g., attention, processing speed) in patients with PD result in gait interference, deficits in emotional processing, loss of functional capacity (e.g., intellectual activity, social participation), and reduced quality of life [2,3]. While research supports the effectiveness of music listening interventions (e.g., Rhythmic Auditory Stimulation) on mobility, balance, and gait [4,5,6,7], the impact of more enactive music experiences (i.e., music training) on deficits in executive functions in PD is not yet known. The use of music training to enhance cognitive outcomes in adults with PD is supported in this rationale.

## 2. Deficits in Executive Functions in PD 

In a meta-analysis of executive function ability in adults with PD, Kudlicka, Clare, and Hindle [8] found significant deficits in all tested skills including cognitive flexibility, set switching, inhibition, selection attention, working memory, and concept formation. These deficits may be compounded by typical age-related cognitive decline and may be the precursor to mild cognitive impairment (PD-MCI) and dementia (PDD) [9]. Yet, despite these cognitive deficits, most interventions focus upon implementing movement for patients with PD and fail to discriminate between differential cognitive profiles. 

Discrepancies in whole-brain functional connectivity may be explained by the different levels of cognitive ability found in patients with PD. Lopes et al. [10] examined four categories of cognitive profiles from 119 patient participants: cognitively intact, those with only slight mental slowing, those with mild to moderate deficits predominantly in executive functions, and those with severe deficits in all cognitive domains. Age and education accounted for, there were significant group neural connectivity differences in each category. Essentially, the main neural areas involved the ventral prefrontal, parietal, temporal, and occipital cortices as well as the basal ganglia. As cognitive levels decrease, the network organization is progressively disrupted, with increased numbers of altered connections between the above-mentioned brain regions. Mak et al. [11] examined structural magnetic resonance imaging of 105 patients with PD and 37 controls at baseline and at 18 months. Those patients with PD without dementia at baseline developed significant frontal cortical thinning over 18 months. The researchers concluded that the increased frontal thinning is associated with concurrent dopaminergic, serotonergic, cholinergic, or noradrenergic frontal–striatal circuit disruptions. 

Deficits in executive functions can lead to mild cognitive impairment, and eventually progress to dementia [12]. Specifically, the longitudinal status of cognitive ability in adults with PD was examined by Pedersen et al. [12] at baseline (*n* = 178), and then at 1 (*n* = 175), 3 (*n* = 163), and 5 (*n* = 150) years. One observed trend was a progression of cognitive decline, regardless of baseline cognitive status. For those with normal cognition at baseline (*n* = 142), the incidence of mild cognitive impairment (MCI) was 9.9%, 23.2%, and 28.9% by year 1, 3, and 5, respectively. While 39.1% of those with baseline or incident MCI progressed to dementia by year 5, a greater percent of those with persistent MCI, 59.1%, progressed to dementia by the fifth year. Of patients with normal cognition at baseline, 7.2% converted to dementia by the fifth year. Still, progressive decline was not definite; the remainder of those with normal cognition at baseline retained their ability. Moreover, 24% of those with incident MCI reverted to normal cognition by year 5. However, the researchers stated that those who reverted to normal cognition within the first three years are at a continued risk for developing dementia. Given these findings, those patients with MCI who revert to normal cognition may be excellent candidates for cognitive interventions to prevent progressive cognitive decline.

## 3. A Theory for Improving Cognition in Adults with PD

Aging is associated with decreases in white matter tracts, resulting in a reduced supply of motor resources when compared to young adults [13]. Consequently, aging adults demonstrate structural and functional reductions in motor-based performance and cognitive resources. As the long white matter tracts degenerate, the neuronal assemblies which link sensory awareness, cognitive drive, emotional efficacy, and motor action de-differentiate. The capacity for differentiated adaptive action is consequently reduced. To optimally re-differentiate remaining resources, compensation can engage the mechanisms of neuronal plasticity.

The cerebellum, an area associated with fine motor movements, may hold the key to improving cognition in aging as explained in part by the Supply and Demand Framework [13]. According to this framework, older adults rely upon cognitive brain processes for motor control, resulting in increased cognitive demand. Specifically, the dopaminergic degeneration observed in aging adults (and especially in patients with PD) leads to increased dependence on prefrontal regions to consciously control motor and cognitive tasks. Thus, rehabilitation interventions targeting motor skills, cognitive skills, or the dopaminergic system may have a shared benefit to other behavioral or neural systems. Successful regulation and maintenance of cognition in aging depends upon coordination and integration between brain areas dominated by a critical neurochemical modulatory system.

The frontal, dopaminergic system is concerned fundamentally with motivated behavior and the capacity to plan and control a course of action and to successfully evaluate, in real time, the consequences of actions. Seidler’s [13] Supply and Demand Framework asserts that the dopaminergic system acts on the cortico-cerebellar neural pathways that project from the cerebellum to the frontal cortices—areas associated with higher-level cognitive processing. Thus, if the cortico-cerebellar pathway is strengthened through fine motor skills in music training, this may result in enhanced cognitive performance or mitigate potential cognitive deficits.

## 4. Neuroplasticity and Cognitive Improvements

Improvements in cognitive functioning may be explained by structural and functional neural plasticity. Neural plasticity is defined as, “the ability of the nervous system to respond to intrinsic and extrinsic stimuli by reorganizing its structure, function, and connections” [14]). An extensive review by Sweatt [15] explains how these neural and biochemical mechanisms have been understood in recent decades. First, behavior may be influenced by synaptic plasticity via long-term potentiation (LTP)—a strengthening of the synapses between neurons. LTP is directly implicated in cognitive functions such as learning and memory as related to neural structures including the amygdala, cerebral cortex, and hippocampus. In contrast to structural plasticity, functional plasticity is associated with Hebbian synaptic plasticity, a theory suggesting that the strength of a neural connection may be dependent upon repeated and persistent shared activity between neurons [16]. 

Neurogenesis, or the development of new and functional neurons, within behavioral circuits is a third mechanism underlying behavioral changes. Finally, experiences can drive the production and regulation of epigenetic molecular mechanisms and impact gene transcription within the CNS. This process impacting the expression of epigenetic marks in adults may underlie enduring changes in behaviors associated with cognition function including memory formation and attention. These cognitive functions contribute to executive functions. 

Current understanding of how cognitive training programs protect older adults from cognitive decline is sparse. Kim et al. [17] point to neuroplasticity as an essential mechanism behind cognitive rehabilitation in several clinical populations and in older adults. However, they point out that many studies implicated in neuroplasticity and learning are limited to simple forms of learning, while the relationship between higher-order cognitive functions and neuroplasticity is not fully understood. The authors provide a theoretical framework for understanding the mechanisms underlying cognitive rehabilitation in older adults. Neural changes induced by cognitive rehabilitation can be generally divided into two categories: (1) stimulation, which induces functional brain reorganization via interaction with external stimuli; and (2) compensation, which involves an adaptive reorganization in response to internal damage or degeneration. Cognitive training may mediate these neural mechanisms. Stimulation-based training may restore functional connectivity across diverse brain regions as targeted by specific cognitive exercises (e.g., attention, working memory, and language). Meanwhile, compensation-based training targeting attention or executive functions may be supported by frontal-mediated adjustments. Thus, mechanisms for neuroplasticity observed in response to cognitive rehabilitation may be explained by multiple neural adaptations.

### 4.1. Neuroplasticity and Music Training

Many researchers suggest that musicians’ brains may serve as models for neuroplasticity [18,19,20,21,22,23]. Neurological data confirm transferability from specific musical skills to a broad range of neural correlates not limited to the motor cortices, superior temporal gyrus, basal ganglia, and cerebellum. For instance, research suggests that fine motor skills implicated in piano training result in increased gray matter density in pianists compared with in nonmusicians [24]. Similarly, results of another study implicating piano training showed an increased positive connectivity in the left primary motor cortical area to the right cerebellum post-training in youth with neurodevelopmental disorders [25]. Data in both of these studies showed enhanced activations in the left primary sensorimotor cortex and right cerebellum. 

In music training, structural and functional neural plasticity have been associated with specific musical instruments [26]. Imaging data confirm that musical experiences in terms of instrument training and the level of practice strengthened connections between auditory and motor regions. The strengthened neurological connections, evident in musicians with long-term music training, show enhanced sensorimotor and cognitive performance. 

### 4.2. Music, Temporal Entrainment, and Cognition 

Temporal entrainment to music via the auditory–motor system is well understood, but less so with cognition. Short-term interventions with temporal elements may improve executive functions in patients with PD [5,27,28]. For instance, rhythmic complexity in tango dancing (18 h over 12 weeks) enhanced spatial cognition compared to education training [29]. The model of temporal prediction and timing suggests that two networks collectively contribute to movement generation and execution: the basal ganglia-thalamocortical network (BGTC) and the cerebellar-thalamocortical network (CTC) [30,31]. Due to decreases in dopamine responsible for the disruption of the BGTC network in PD, it is hypothesized that rhythmic auditory cues, like those used in piano training, assist patients through recruiting an alternate CTC network which extends to the supplementary motor areas (SMA) and the frontal cortices [31,32]. In piano training, fine rhythmic motor finger movements that activate the cerebellum and SMA essentially exercise the CTC network [31,33]. 

Additionally, evidence from temporal entrainment in music mnemonic training of learning and recall of word lists has shown changes in brain plasticity of oscillatory neural networks [34]. The music-induced plasticity, indicated by higher electroencephalography synchrony in learning-related networks, was concomitant with significantly better recall during music (sung) versus spoken recall. The improved word recall performance was found in both healthy adults and those with multiple sclerosis, a condition known for deficits in executive functions, similar to those seen in adults with PD.

### 4.3. Music Training and Healthy Adults with Age-Related Cognitive Decline 

Music training includes complex fine motor skills that rely upon sensorimotor processing. Music training can transfer to a broad range of cognitive and learning domains and may serve as an effective cognitive training intervention for older adults. The *Model of Successful Aging and Music Participation* suggests that musical performance modulates psychological, physiological, and emotional health in older adults through necessary components critical for cognitive training programs: task novelty, progressive difficulty, sensorimotor integration, practice requirements, and social components [35]. This model has many implications for the benefits of music training in patients with Parkinson’s disease. For instance, learning a new musical skill necessitates the facilitation of increased attention and concentration, believed to exercise areas of executive functions [36]. Task novelty contributes to attentional demands. Sustained attention and selective attention have been shown to contribute to enhanced performance in child musicians when compared with nonmusicians on measures of executive functions [37]. These attentional benefits may be extended into adulthood for those engaging in the highly novel experience of learning a musical instrument. 

Age-related cognitive deficits of 60–85-year-old naïve musicians, namely, abilities found in working memory and planning, were significantly improved with piano instruction [38]. The individualized piano instruction required high levels of temporal and spatial processing, requiring the participant to plan, organize, and sequence a cohesive musical event [38]. Increased reliance upon sustained attention was attributed to enhancements in working memory and processing speed in older adults who received piano training compared to those who did not receive piano trainng. This study is important for the present rationale in that it shows evidence of general executive function enhancement, in contrast to solely music executive function skill. These benefits are particularly important to extend to and investigate in the PD population. A recent systematic review [39] of the efficacy of controlled and noncontrolled studies of cognitive rehabilitation, physical rehabilitation, exercise, and brain stimulation techniques for patients with PD found that interventions should target those with no significant cognitive impairment, those with MCI, or those with dementia. 

In patients with PD, it is even more critical to receive opportunities to exercise executive functions to mitigate further decline. Patients with PD exhibit differentiating levels of deficits in executive functions. Compensation for such deficits is evident in patients in the moderate stages of the disease [40]. Compensation strategies include planning for cognitive tasks to account for limitations in psychomotor speed. Interventions such as music training can facilitate increases in motor and cognitive processing speed through practice of progressively difficult passages requiring neurological processing of rhythmic and sequential musical structures. Further, music therapy for adults with PD enhances their ability to perceive rhythm in rhythmic auditory cuing, a rhythm-based treatment that improves gait in PD [41]. Fine motor training may facilitate a different layer of complexity in music education programs with progressive difficulty. As such, these programs have the potential to activate cerebellar structures contributing to neuroplasticity.

### 4.4. Music and Adults with Deficits in Executive Functions 

Cognitive flexibility, a higher-order executive function, was significantly improved in adults with executive function deficits (e.g., stroke, traumatic brain injury) following one neurologic music therapy session [42]. While not a music training session per se, the adults participated in instrument playing and made decisions about their group improvisation. Exercising decision-making, planning, and problem-solving through facilitated music experiences constitutes the neurologic music therapy technique referred to as music executive function training (MEFT) [43]. Music training that emphasizes these executive functions as goal areas may also be referred to as MEFT. Indeed, feasibility and intervention criteria for MEFT successfully employed to address task-shifting in adults with acquired brain injury are outlined by Lynch and LaGasee [44]. Music training that involves attainable incremental challenges, novelty, emotional arousal, and exercises task-shifting, sequencing, and so forth may also be of benefit to the executive function needs of adults with PD. 

Therapeutic music activities have successfully targeted and improved executive functioning in adult clinical populations. “Chemo-brain,” a frequent side effect of chemotherapy treatments, often produces attention and memory problems. Fifteen women receiving adjuvant chemotherapy for breast cancer received individualized once-weekly mindfulness-based music therapy, along with daily homework, for four weeks. While not music training per se, the women learned to play simple patterns on percussion and harmonic instruments (e.g., xylophone, piano) as part of the mindfulness-based music therapy four-week program. Information processing speed, as measured through a standardized computer test, was significantly improved, and, as well, symptom distress was significantly decreased [45]. The resulting cognitive improvement may be explained by the stimulation of the music program as opposed to a compensatory mechanism as mentioned by Kim et al. [17].

## 5. Conclusions and Recommendations 

Together, this evidence presents a compelling case for music training to improve executive functioning for adults with PD. First, music training activates the cerebellar-thalamocortical network (CTC) network providing a rerouting to activate executive functions through fine motor activity [33]. We hypothesize that exercising the CTC network through music training will contribute to enhanced cognitive performance. The Movement Disorder Society (MDS) values the identification and intervention of cognitive impairment in adults with PD, and sees it as part of essential care—a need yet to be met [46]. Cognitive interventions that include repeated practice exercising the CTC network through sensorimotor integration may assist patients with PD. While research has shown that music training enhances cognitive performance (i.e., working memory and processing speed) in healthy older adults [38,47,48], there is a need to extend the benefits of music training to patients with PD. It will be necessary to fully ascertain the benefits of music training through large-scale randomized controlled trials. Researchers, patients, and caregivers would greatly benefit from published protocols that provide clear descriptions and manuals for musical interventions. Music training, as a type of cognitive rehabilitation, should also adhere to these guidelines and provide an evidence base for its use for adults with PD. 
